# Patient-reported outcomes of serum eye drops manufactured from Australian blood donations and packaged using Meise vials

**DOI:** 10.3389/fmed.2023.1252688

**Published:** 2023-09-05

**Authors:** Carley N. Gemelli, Phillip Mondy, Athina Kakkos, Justine O’Donovan, Perfecto Diaz, Elizabeth Knight, Rena Hirani

**Affiliations:** ^1^Australian Red Cross Lifeblood, Melbourne, VIC, Australia; ^2^Australian Red Cross Lifeblood, Sydney, NSW, Australia; ^3^Faculty of Science and Engineering, Macquarie University, Sydney, NSW, Australia

**Keywords:** blood donation, serum eye drops, transfusion medicine, dry eye, blood

## Abstract

**Introduction:**

Serum eye drops (SED) are an effective treatment for dry eye syndrome. However, autologous serum collection can have challenges. Patient-tailored (allogeneic) SED (PT-SED) can be made from healthy blood donors. Australian Red Cross Lifeblood has manufactured both autologous SED (Auto-SED) and PT-SED and, in May 2021, introduced Meise vial packaging. This study aimed to explore SED patient-reported outcomes and vial packaging satisfaction.

**Methods:**

A prospective cohort study was conducted with recruitment between 1 November 2021 and 30 June 2022. Participants completed the dry eye questionnaire (DEQ5), health-related quality-of-life (SF-8^™^), functional assessment of chronic illness therapy-treatment satisfaction-general (FACIT-TS-G), and general wellbeing surveys. Existing patients completed these once, and new patients were surveyed at baseline, 3 months post-treatment, and 6 months post-treatment.

**Results:**

Participants who completed all study requirements were 24 existing and 40 new Auto-SED and 10 existing and 8 new PT-SED patients. Auto-SED patients were younger [56.2 (±14.7) years] than PT-SED patients [71.4 (±10.0) years]. Participants used a mean of 1.8 (±1.1) SED, 5.3 (±2.9) times per day. In new patients, DEQ5 scores improved within 6 months from 14.0 (±2.9) to 10.6 (±3.4) for Auto-SED and from 12.9 (±3.7) to 11.4 (±2.8) for PT-SED. General wellbeing measures improved in the new Auto-SED from 7.0 (±1.9) to 7.8 (±1.7) but were reduced for new PT-SED from 6.7 (±2.9) to 6.1 (±2.9).

**Discussion:**

SED improved dry eye symptoms in most patients, regardless of the serum source. Patients using PT-SED showed decreases in some quality-of-life measures; however, recruitment was reduced due to operational constraints, and concurrent comorbidities were not assessed. General feedback for SED and vial packaging was positive, with some improvements identified.

## 1. Introduction

Severe dry eye disease (also known as dry eye syndrome or keratoconjunctivitis sicca) is a commonly diagnosed condition and, within a summary of international prevalence, is noted to affect up to 50% of patients referred to ophthalmologists (1). Dry eye is caused by the inability to produce enough tears for lubrication or tear evaporation, resulting in severe quality of life challenges for the affected person (2, 3). Dry eye is mostly found in older women and has a range of potential causes, including hormonal changes; medical conditions, such as autoimmune conditions or rheumatological diseases; medications; and environment. However, depending on the definition of dry eye that is used, it is becoming more commonly reported and is linked with the use of computers and other screen technologies (4, 5). In Australia, vision problems are reported to affect 9.4% of Australians aged over 55 years (6).

There are a number of available treatments and surgical interventions, such as preservative-free ocular lubricants, punctal occlusion, night-time ointment or moisture goggles, therapeutic contact lenses, topical anti-inflammatory medications (corticosteroids and cyclosporine), or oral antibiotics (macrolide or tetracycline). However, one well-tolerated treatment is serum eye drops (SED), which are reported to have few side effects, including slight eye irritation, burning, and tearing with the potential for an increased risk of infection if the eye drop vessel is not handled as directed (7–16).

SED are made by separating the serum from the cellular components of whole blood (WB) and can be made from a patient’s own blood donation (autologous) or using blood donations from healthy volunteers (allogeneic). SED manufacturing procedures are not standard internationally and differ between blood collection agencies, pathology clinics, and other providers. SED manufacture can differ in the amount of WB collected, concentration, type of diluent, and packaging systems (17). These differences can make effective comparisons of patient outcomes following the use of SED more challenging and impact the understanding of which proteins or growth factors are vital for the most effective SED composition.

In Australia, Australian Red Cross Lifeblood (Lifeblood) is the national provider of fresh blood and blood products manufactured from blood donations made by voluntary, non-remunerated donors. Currently, Lifeblood manufactures both autologous serum eye drops (Auto-SED) and patient-tailored (allogenic) serum eye drops (PT-SED) diluted to a concentration of 20% in 0.9% saline. Following manufacture, SED components are transported using dry-ice to an approved health provider (AHP) close to the patient’s home, and patients are notified to collect them. Auto-SED patients receive a 12 months supply, and PT-SED patients receive a 6 months supply, which are stored by the patients in domestic freezers. Both sources of SED have a 12 months expiration date following manufacture. Once opened, SED users are asked to dispose of vials at the end of the day.

For SED provision, patients must have a referral from a consultant ophthalmologist where there is a reasonable expectation of therapeutic benefit. Suitability for Auto-SED collection is dependent on the patient meeting general blood donation eligibility criteria, having reasonable venous access, and having the ability to tolerate venesection. Prior to the COVID-19 pandemic, blood collection for Auto-SED was conducted via a Lifeblood blood donation centre or, in particular cases, using selected AHPs. PT-SED are a desirable alternative for cases where patients are unable to donate due to poor venous access, have comorbidities preventing donation, or have mobility/geographical restrictions, and they also enable the ability to streamline manufacturing processes (18). As a result of the COVID-19 pandemic in 2020, health service restrictions prevented the collection of autologous donations from patients within AHPs, and PT-SED production increased.

**Figure 1:**
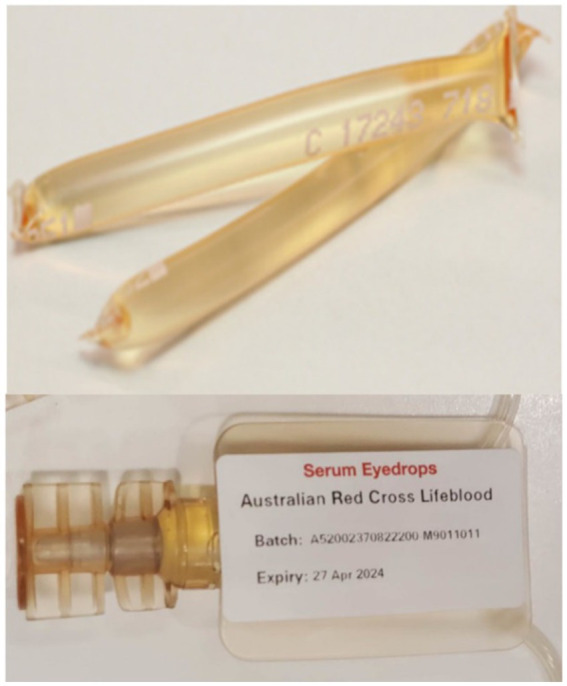
Photograph of the original tubing segments (top) used for serum eye drops and the Meise vials (bottom) introduced in May 2021.

Furthermore in May 2020, Lifeblood introduced Meise vial (Meise Medizintechnik GmbH) packaging for SED (Figure 1). Prior to this, SED were packaged using segmented plastic tubing (Macopharma VSE4001XK tubing set) (19). The introduction of closed vials is known to improve efficiencies in SED manufacture and assist patients in the administration of SED onto the ocular surface (20). The previous segments were reported to be difficult to open, as they had to be cut open with scissors and were difficult to squeeze to get the drops out. They were also labour-intensive to manufacture, as they required manual heat sealing to produce a single-dose segment length (17). The vials are easier to open, as they have a twist cap, can be recapped and stored for the day, and are already segmented into vials, making them more efficient to manufacture. Furthermore, using vial packaging did not affect the stability of SED composition (19, 20).

Previous studies have shown that Auto-SED has sustained benefits for dry eye in Australian patients (14). However, there are limited standardised studies on the effectiveness of comparing Auto-SED and PT-SED, especially for patients who have been swapped between SED types. One Netherlands study did indicate that autologous and allogeneic SED have comparable efficacy and tolerability (18).

In this study, we explore both Auto-SED and PT-SED patient group perspectives on the effectiveness of SED products on their eye symptoms and quality of life using standardised dry eye and quality of life surveys. Views from all SED patients were also explored on the vial packaging.

## 2. Methods

The study was reviewed and approved by the Australian Red Cross Lifeblood Human Research Ethics Committee (HREC) (Mondy 21092021). The study was conducted in accordance with the National Health and Medical Research Council’s National Statement on Ethical Conduct in Human Research (2007, updated 2018).

### 2.1. Patient enrolment

Eligible SED patients between 1 November 2021 and 30 June 2022, over 18 years of age, who were able to provide written informed consent and were not current Lifeblood staff members, were identified through the National Blood Management System (NBMS) administered by Lifeblood. Potential participants were screened by a research study nurse to determine study eligibility. Once eligible, patients were invited to participate in the study via email or postal letter. Following participant consent, links to the survey were sent, and data were collected via the online web platform Qualtrics (Qualtrics, Provo, UT). If a valid email address was not available or if the participant requested it, paper-based surveys were sent to the recorded postal address. Participants were also able to complete the surveys with research team assistance over the telephone if they requested so. When there was no response, participants received a reminder email within 10 days. Follow-up telephone calls were also conducted if no response had been received 2 weeks after initial contact. If participants asked for the survey to be completed via telephone, a research staff member would read out the questions and complete the form on Qualtrics. Any feedback provided outside of the survey questions was not recorded to ensure that the feedback was the same as for participants who completed the survey online or via paper-based copies.

Auto-SED and PT-SED participants were divided into two groups: those receiving the product for the first time were classified as “New,” while those who had received SED before this research study were classified as “Existing.” The groups were identified before the participant was invited to ensure that the correct survey tools were provided. All patient research records were managed using REDCap (Research Electronic Data Capture), a secure web platform supporting data capture for research studies (21). A research study nurse verified details regarding patient medications and clinical indications.

### 2.2. Survey and interview

Existing patients received only a single survey sent at the time when their next allotment of SED was available. New patients received surveys at baseline prior to SED treatment to establish a pre-treatment measure. They then received two follow-up surveys at 3 months and 6 months post-SED commencement. Survey questions were removed or included depending on the timepoint as required to comment on SED usage where appropriate. Brief outlines of each survey section are provided below, with a detailed survey questions provided in Supplementary Figure S1. The survey tools used were chosen because they are well-reported, simple, and objectively standardised measurement tools within the medical field to measure patient-reported outcomes. They were chosen to determine not only whether dry eye symptoms were reduced but also whether the relief of these symptoms assisted patients with other general wellbeing measures and provided insight into the wider health status of this patient group.

### 2.3. Dry eye questionnaire

The dry eye questionnaire (DEQ5) assesses a patient’s experience of dry eye symptoms on a typical day over a month. Questions were included to ascertain the severity and degree of eye discomfort, eye dryness, and excessive wateriness. The five measures are combined into a total score ranging from 0 to 22, where the lower the score, the less severe the symptoms (22).

### 2.4. Short form health survey

The short form health survey (SF-8^™^) is a shortened version of the SF-36^™^ health survey and provides a generic assessment of health-related quality of life in adults, including physical health and functioning, role limitations, bodily pain, vitality, social functioning, mental health, and emotional challenges (23, 24). Scores range from 0 to 100, with higher totals indicating better health.

### 2.5. General wellbeing

The National Eye Institute visual function questionnaire (NEI-VFQ-25) captures vision and health-related quality of life and is one of the most commonly implemented patient-reported outcomes in ophthalmology research (25). For this study, two items from the NEI-VFQ-25 have been used to describe the current level of wellbeing and distress. Specifically, these are “I am often irritable because of my eyesight” and “I do not go out of my home alone, because of my eyesight.” Scores ranged from 2 to 10, with a higher score indicating a greater level of wellbeing experienced.

### 2.6. Functional assessment of chronic illness therapy-treatment satisfaction-general

The functional assessment of chronic illness therapy-treatment satisfaction-general (FACIT-TS-G) version 4 measures a patient’s satisfaction with the treatment they have been administered (26). Patients are asked to rate the effectiveness of the treatment compared to expectations, satisfaction with the treatment, physician evaluated effects of the treatment, whether they would use the treatment again, and recommendations to others. The responses are combined to provide a score ranging from 0 to 25, with a higher score indicating more satisfaction with the treatment.

### 2.7. Generic SED packaging and satisfaction questions

SED treatment-related questions, frequency and volume of SED use, and views on SED were also asked and are outlined in Supplementary Figure S2. Text from open fields within the survey was analysed and coded for inclusion in relevant themes.

### 2.8. Qualitative interviews

Opt-in participation in a 30 min semi-structured, in-depth phone interview was also conducted on up to five selected individuals per SED group (see Supplementary Figure S2 for a detailed question list). The participants were invited to a qualitative interview at the time of enrollment until up to five individuals had been interviewed per group. Topics included views on SED packaging, SED usage, and the impact on quality of life. The interviews were recorded with consent and transcribed by a contracted transcription service. Participants received a token gift card to the value of AUD50 to recompense for their time. Interview transcripts were analysed using an inductive method, with some sections of the transcript coded.

### 2.9. Statistical method

Statistical analyses of the quantitative data were performed using the statistical software IBM SPSS (IBM SPSS Statistics 28.0; IBM Corporation). Demographic and donation characteristics were described by means (±SD) for continuous parametric variables, medians (Med) [interquartile ranges (IQR)] for non-parametric variables, and by totals (percentages) for categorical variables. Independent *t*-tests were conducted to determine any univariate means differences between the timepoints for parametric data, and a Wilcoxon signed-rank test was used for non-parametric variables. Statistical significance was determined as a two-tailed *p*-value of ≤0.05.

## 3. Results

### 3.1. Patient demographics and dry eye diagnosis

**Figure 2:**
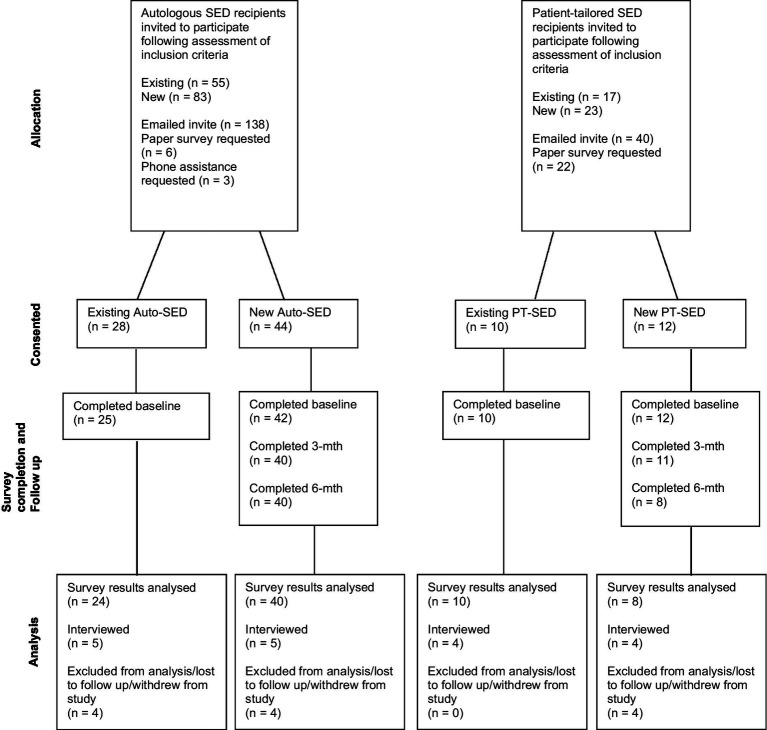
Consort diagram of patient enrolment and completion of study requirements.

The number of patients who were provided SED during the study period was 186, of which 144 were Auto-SED recipients and 42 were PT-SED recipients. Six Auto-SED and two PT-SED patients did not meet the study requirements; therefore, a total of 178 potential participants were invited to participate in the study. Of these, 138 (55 existing and 83 new) were autologous recipients (74.2% of total patients provided with Auto-SED), and 40 (17 existing and 23 new) were patient-tailored (allogenic) recipients (95.2% of total patients provided with PT-SED) (Figure 2).

Of those invited, a total of 28 existing (50.9% of existing invited) and 44 new (53.0% of new invited) Auto-SED patients and 10 existing (58.8% of existing invited) and 12 new (52.2% of new invited) PT-SED patients consented to participate in the study. However, participants who completed all study requirements for analysis consisted of 24 existing (43.6% of existing invited) and 40 new (48.2% of new invited) Auto-SED recipients (44.4% of total patients provided with Auto-SED), 10 existing (58.8% of existing invited), and 8 new (34.8% of new invited) PT-SED patients (42.9% of total patients provided with PT-SED). Previous autologous SED patients that were now being provided with PT-SED due to operational and manufacturing reasons were combined with existing PT-SED patients to enable a more robust analysis.

Table 1 summarises the demographic and clinical indications for SED requirements. The mean age of existing Auto-SED patients was 58.0 (±12.2) years and 55.1 (±16.0) years for new Auto-SED patients. PT-SED patients were older than Auto-SED patients, with a mean age of 70.7 (±11.5) years for existing patients and 72.3 (±8.7) years for new patients. Most (82.8% for Auto-SED and 94.4% for PT-SED) of the patients requiring SED were women. SED treatment was required due to the diagnosis of Sjögren’s syndrome (51.6 and 61.1% for Auto-SED and PT-SED, respectively). SED were also required for neurotrophic corneal diagnosis in 17.2% of Auto-SED patients. Dry eye symptoms were experienced for approximately 5 years or more, and nearly all patients (82.8% Auto-SED and 88.9% PT-SED) had tried at least one other treatment prior to SED. Existing Auto-SED patients had symptoms longer than new Auto-SED patients [15.4 (±14.7) years and 4.9 (±5.5) years, respectively]. Existing PT-SED patients had symptoms for a shorter time than new PT-SED patients [10.0 (±13.5) years and 23.3 (±17.3) years, respectively].

**Table 1:** Demographics of consented serum eye drop users enrolled.

		Autologous patients		Patient-tailored	
		Existing (*n* = 24)	New (*n* = 40)	Existing (*n* = 10)	New (*n* = 8)
Age (mean ± SD)		58.0 (±12.2)	55.1 (±16.0)	70.7 (±11.5)	72.3 (±8.7)
Gender [*n* (% of cohort total)]	Male	4 (16.7)	7 (17.5)	0 (0.0)	1 (12.5)
	Female	20 (83.3)	33 (82.5)	10 (100.0)	7 (87.5)
Prior auto donations (mean ± SD)		4.0 (±3.7)	0 (0.0)	—	—
Clinical indications resulting in dry eye syndrome [*n* (% of the cohort total)]^a^					
Acute management of corneal injuries		2 (8.3)	1 (2.5)	0 (0.0)	0 (0.0)
Blepharitis and conjunctivochalasis		0 (0.0)	1 (2.5)	0 (0.0)	0 (0.0)
Graft vs. host		1 (4.2)	0 (0.0)	0 (0.0)	1 (12.5)
Inherited ocular surface disease		1 (4.2)	6 (15.0)	0 (0.0)	0 (0.0)
Neurotrophic cornea		5 (20.8)	6 (15.0)	0 (0.0)	2 (25.0)
Ocular mucous		0	1 (2.5)	0 (0.0)	0 (0.0)
Sjögrens syndrome		10 (41.7)	23 (57.5)	7 (70.0)	4 (50.0)
Stevens–Johnson syndrome		2 (8.3)	1 (2.5)	0 (0.0)	0 (0.0)
Supportive		0	2 (5.0)	0 (0.0)	1 (12.5)
Other		4 (16.7)	2 (5.0)	3 (30.0)	2 (25.0)
Number of years with symptoms (mean ± SD)		15.4 (±14.7)	4.9 (±5.5)	10.0 (±13.5)	23.3 (±17.3)
Currently or previously used at least one other treatment [*n* (% of cohort total)]^a^		22 (91.7)	31 (77.5)	8 (80.0)	8 (100.0)
Artificial tears		19 (79.2)	26 (65.0)	7 (70.0)	7 (87.5)
Topical anti-inflammatory drops		17 (70.8)	27 (67.5)	5 (50.0)	6 (75.0)
Tear retention therapies		16 (66.7)	23 (57.5)	6 (60.0)	2 (25.0)
Other		15 (62.5)	18 (45.0)	6 (60.0)	1 (12.5)

a Multiple selections could have been made.

### 3.2. Survey measures

DEQ5 measures were found to change significantly in new Auto-SED patients from baseline to 6 months post-treatment [14.0 (±2.9) to 10.6 (±3.4), *p* < 0.001] (Table 2). Although not statistically significant in new PT-SED patients, the DEQ5 scores trended down from baseline to 6 months post-treatment [12.9 (±3.7) to 11.4 (±2.8)], indicating improvements for some patients. DEQ5 scores were similar for existing Auto-SED and existing PT-SED at 12.7 (±2.7) and 12.9 (±3.3), respectively.

**Table 2:** Survey score totals from each group of serum eye drop (SED) users.

Survey (mean ± SD)	Autologous serum eye drop patients						Patient-tailored serum eye drop patients					
	Existing (*n* = 24)	New (*n* = 40)	New 3 months post-SED	Change when compared to baseline (*p*-value)^a^	New 6 months post-SED	Change when compared to baseline (*p*-value)^a^	Existing (*n* = 10)	New (*n* = 8)	New 3 months post-SED	Change when compared to baseline (*p*-value)^a^	New 6 months post-SED	Change when compared to baseline (*p*-value)^a^
DEQ5 discomfort	3.0 (±0.8)	3.4 (±0.7)	2.8 (±0.8)	<0.001^a^	2.4 (±0.8)	<0.001^a^	3.2 (±1.0)	2.8 (±1.3)	2.3 (±1.2)	0.45	2.8 (±0.7)	1.00
DEQ5 intensity of discomfort	2.8 (±0.8)	3.1 (±0.8)	2.4 (±1.1)	<0.001^a^	2.4 (±1.0)	<0.001^a^	2.9 (±1.0)	2.7 (±1.0)	2.0 (±1.1)	0.14	2.0 (±1.4)	0.23
DEQ5 dry	3.0 (±1.0)	3.3 (±0.8)	2.7 (±0.9)	<0.001^a^	2.6 (±0.8)	<0.001^a^	2.7 (±1.4)	2.9 (±1.1)	2.1 (±0.8)	0.08	2.6 (±0.7)	0.45
DEQ5 intensity of dryness	3.0 (±0.7)	3.1 (±0.9)	2.4 (±1.1)	<0.001^a^	2.3 (±0.9)	<0.001^a^	2.9 (±1.4)	3.0 (±0.9)	2.0 (±1.3)	0.07	2.4 (±1.2)	0.18
DEQ5 watery	1.0 (±0.9)	1.1 (±1.1)	1.3 (±1.2)	0.42	1.0 (±1.1)	0.28	1.2 (±1.4)	1.3 (±1.3)	1.1 (±1.5)	0.83	1.6 (±1.3)	0.40
DEQ5 total	12.7 (±2.7)	14.0 (±2.9)	11.5 (±3.7)	<0.001^a^	10.6 (±3.4)	<0.001^a^	12.9 (±3.3)	12.9 (±3.7)	9.5 (±5.0)	0.10	11.4 (±2.8)	0.32
SF-8^™^ total	19.5 (±8.1)	19.6 (±6.7)	19.2 (±6.8)	0.59	18.7 (±6.0)	0.33	26.1 (±6.6)	26.3 (±9.5)	25.4 (±9.3)	0.68	29.3 (±7.7)	0.39
General wellbeing total	7.1 (±2.1)	7.0 (±1.9)	7.5 (±2.0)	0.10	7.8 (±1.7)	0.002^a^	6.4 (±2.3)	6.7 (±2.9)	7.3 (±2.8)	0.92	6.1 (±2.9)	0.36
FACIT-TS-G total	21.5 (±3.8)	—	16.4 (±5.8)	—	17.6 (±5.3)	0.05^a^	19.2 (±4.6)	—	20.1 (±4.1)	—	18.4 (±4.6)	0.66

DEQ5, dry eye questionnaire (DEQ5); SF-8^™^, 2.4 short form health survey; FACIT-TS-G, functional assessment of chronic illness therapy-treatment satisfaction-general.

a Two-tailed *p*-value ≤0.05 is considered significant.

In new Auto-SED patients, the SF-8^™^ survey showed no improvement from baseline to 6 months post-treatment [19.6 (±6.7) to 18.7 (±6.0)]. In new PT-SED patients, the SF-8^™^ was slightly improved at 6 months post-treatment, but this was not significant [26.3 (±9.5) to 29.3 (±7.7), *p* = 0.39]. Overall, for existing patients, the SF-8^™^ score was higher for PT-SED users than for Auto-SED users.

General wellbeing improved for new Auto-SED users 6 months post-SED treatment [7.0 (±1.9) to 7.8 (±1.7), *p* = 0.002] but not for new PT-SED users [6.7 (±2.9) to 6.1 (±2.9), *p* = 0.36]. Overall, the wellbeing score was higher for existing Auto-SED patients than for existing PT-SED patients [7.1 (±2.1) and 6.4 (±2.3)].

The FACIT-TS-G surveys did show a slight improvement in new Auto-SED patients 6 months post-treatment [16.4 (±5.8) to 17.6 (±5.3)] but was not improved in new PT-SED patients [20.1 (±4.1) to 18.4 (±4.6)]. Overall, the FACIT-TS-G surveys were similar between existing Auto-SED and existing PT-SED patients [21.5 (±3.8) and 19.2 (±4.6)].

### 3.3. Serum eye drop usage and patient comments

The number of times SED were used per day and the number of drops each time were similar between Auto-SED and PT-SED users with approximately two drops used up to six times per day (Table 3). Notably, 1 to 1.5 vials were used per day. Up to four vials were discarded due to damage. Up to 180 vials were reported to be discarded due to expiration dates being reached.

**Table 3:** Summarised results of serum eye drop (SED)-related use and discard.

SED-related outcome (mean ± SD)	Autologous serum eye drop patients			Patient-tailored serum eye drop patients		
	Existing (*n* = 24)	New 3 months post-SED (*n* = 40)	New 6 months post-SED	Existing (*n* = 10)	New 3 months post-SED (*n* = 8)	New 6 months post-SED
Use per day (times)	5.2 (±2.2)	5.5 (±3.2)	5.3 (±4.1)	6.0 (±3.8)	3.9 (±1.7)	4.5 (±2.5)
How many drops each time	2.1 (±1.1)	1.7 (±1.0)	1.9 (±1.7)	2.1 (±1.5)	1.7 (±1.0)	2.1 (±1.3)
How many vials used per day	1.5 (±1.0)	1.0 (±0.3)	1.1 (±0.4)	1.5 (±0.8)	1.5 (±0.5)	1.1 (±0.4)
How many vials disposed of due to damage	1.3 (±1.0)	2.3 (±1.9)	4.0 (±2.2)	1.0^a^	0.0^a^	2.0^a^
How many vials disposed of due to expiry	37.6 (±27.0)	8.0 (±0.0)	5.0 (±2.8)	180.0^a^	3.0^a^	0.0^a^

a Only one patient provided discard information.

Many patients indicated positive sentiments towards their SED treatment, with many comments indicating that they were considered “life-changing.”

“*It helps me quite a lot because I have a lot of eye complications, so the eyedrops really help my eyes feel comfortable. Because I had such dry eyes that, when I would blink, it felt like there were rocks in my eyes, that’s how weird and dry they were but the eyedrops really, really help. In fact, I have scarring on my corneas, and it turns out that the serum eyedrops seem to have helped heal that scarring, just a little bit, so that’s been very pleasant*.”

“*Prior to using the drops my morning vision for up to 3 h after waking was poor. About 3 weeks after commencing the drops (miraculously) my morning vision was restored to normal. This has had a huge positive impact on my life. In addition, my vision generally has improved such that I have less need for reading glasses. Truly, these drops have helped me enormously and I am very grateful*!”

“*Serum drops have been life-changing for me. I have multiple autoimmune conditions and in the last year or more I have reacted badly to every eyedrop that I tried*… *both over the counter and prescription. (I also react badly to any oral medications I have to use for my dry mouth). With the serum drops, all the nasty symptoms of dry eye have eased. I am extremely grateful. Thank you*!”

The packaging was well liked, and most feedback was to provide options to help patients travel with the SED while frozen. Due to natural disasters in Australia, some patients reported that storing SED during that time was challenging.

*The new packaging, which we have had*—*I do not know*—*the last year, or maybe two; it’s really great. It’s much easier to use than the old straw*… *(When asked what they liked) That I do not have to sterilise them. And that you can just snap the top off and use them, put the lid back on and you are done*.

*If I go out somewhere, you cannot take them (Meise vials) with you. You cannot take them with you without going to a lot of trouble, so I just take over-the-counter eye drops to see me through during the day*.

… *I just find when I have used them at room temperature, I just find them a bit ineffective, so I figure, well I’m not going to (take them out)—it’s too problematic to travel with them that they need to stay frozen, so no*… *Even just outings I will not take them with*.

*For overseas I have to get a licence to travel with (SED)—because it’s human blood, you have to get a special clearance for that, and I did not know about that. So*… *I was unable to travel with them because I did not get the clearance in time*… *I just did not know to travel with them because I did not know how to keep them cold on the flight*… *When I called up my airline, they told me that I needed a special clearance*.

*I got flooded in February, and I had one box left at home—I had two boxes at work, luckily—and I took them to the evac(uation) centre and they had a freezer there that was on a generator, because there was no power*… *By the time I got to the next place they’d all thawed out, but I refroze them and I used them anyway, and I did not have any problems. It’s because I was desperate*… *(My eyes were) really badly irritated from having no sleep and being—probably rubbing them, after being in the funky flood water. They were the worst I’ve ever had them*…

## 4. Discussion

This study aimed to determine the effectiveness of serum eye drops in Australian recipients using standardised surveys and evaluate the patient experience with a vial packaging system that had been recently implemented. DEQ5 was an effective tool for measuring dry eye symptoms in patients and we found that for all patients their dry eye symptoms had reduced in severity after using SED, regardless of whether Auto-SED or PT-SED were used. Only two (2.0%) patients across both cohorts could not use SED or had adverse reactions. This finding is similar to previously published studies on SED related outcomes and appears to be consistent with whether autologous or allogeneic SED were administered (9, 11, 12, 14, 18, 27).

DEQ5 is a reliable and quick tool to measure SED impact on dry eye, and we feel this could be routinely used for monitoring effectiveness in patients who are new to SED treatment (22). The other survey tools analysing health, wellbeing, and distress were not as suitable for assessing SED outcomes as there were disparate responses across users. Disparate findings were likely due to PT-SED patients being significantly older than patients using Auto-SED. Therefore, PT-SED patients may have more associated comorbidities, in addition to dry eye syndrome, that affect their overall wellbeing. Information on associated comorbidities and symptom burden for SED users during the study period was not obtained; therefore, it was challenging to compare the wellbeing outcomes associated with SED to those that were extraneous.

Strength of this study was the use of objective, standardised surveys and following some patients from before SED was applied to 6 months post-treatment. For many SED users, improvements were gained within 3 months of SED use. Limitations included the lack of information on comorbidities that may have impacted the overall wellbeing of the patients. While information on co-treatments was collected, the regime of these treatments when used in conjunction with SED was not provided in detail and would not be useful for any future studies. There was also limited recruitment of PT-SED users as a result of regulatory submissions and operational constraints following the SARS-CoV-2 pandemic impacting SED production during the study recruitment period. Further to disruptions caused by SARS-CoV-2, the study period was additionally impacted by large geographical areas of Australia experiencing extreme weather events, such as flooding, that impacted SED users’ distress and wellbeing. Data biases from telephone interviews were reduced where possible by using standardised tools and questions. However, some variations are possible, especially in older patients, where they may be more positive when they are able to discuss the survey questions with a researcher. Existing patients were used to having SED, so their views on effectiveness may have been diminished. However, their views on the new vial packaging are particularly valuable, as these patients would have been exposed to both the new vials and had used segmented tubing previously. We chose to assess these patients in conjunction with new SED users, to determine whether the vial packaging was preferred.

The use of vial packaging was strongly supported, but there were challenges identified. Some indicated that too much serum volume was being discarded from daily vials as the vial volumes were larger than the previously used segmented tubing. Some patients indicated challenges with opening the vials due to arthritis and other comorbidities affecting fine motor skills. Other patients were frustrated by the lack of solutions to allow travel while keeping the vials at the appropriate cold storage requirements. However, despite these, the vials were strongly supported, and overall use of SED, whether Auto-SED or PT-SED, was seen positively by the patients.

Further to the limited information on patient-reported outcomes following SED use, there is also limited information on whether donor factors affect batches of SED that could influence the effect on patients (19, 28). Despite this, SED, whether autologous or patient-tailored, have been shown to be effective and generally well-tolerated, confirming the findings of other studies. Using tools such as DEQ5 routinely to allow regular standardised measures of associated symptoms and track sustained improvements can be valuable. Overall, this cohort of SED users indicated that their dry eye symptoms were significantly reduced regardless of the source of the drops provided and that vial packaging did improve the patient treatment experience.

## Data availability statement

The raw data supporting the conclusions of this article will be made available by the authors, without undue reservation.

## Ethics statement

The studies involving humans were approved by Australian Red Cross Lifeblood Human Research Ethics Committee. The studies were conducted in accordance with the local legislation and institutional requirements. The participants provided their written informed consent to participate in this study.

## Author contributions

PM conceptualised the study. CG, AK, JO’D, and PD performed project administration, data collection, data analysis, and study interviews. RH supervised the study, analysed data, designed tables/figures, and wrote the manuscript. CG, PM, AK, JO’D, PD, EK, and RH contributed to data interpretation, literature searches/review, study protocol writing, study design, review of the manuscript, and had access to the data to assess and verify study results. All authors contributed to the article and approved the submitted version.

## Funding

Australian governments fund Australian Red Cross Lifeblood to provide blood, blood products and services to the Australian community.

## Conflict of interest

The authors declare that the research was conducted in the absence of any commercial or financial relationships that could be construed as a potential conflict of interest.

## Publisher’s note

All claims expressed in this article are solely those of the authors and do not necessarily represent those of their affiliated organizations, or those of the publisher, the editors and the reviewers. Any product that may be evaluated in this article, or claim that may be made by its manufacturer, is not guaranteed or endorsed by the publisher.

## References

[1] Stapleton F Alves M Bunya VY Jalbert I Lekhanont K Malet F. TFOS DEWS II epidemiology report. Ocul Surf. (2017) 15:334–65. doi: 10.1016/j.jtos.2017.05.003, PMID: 28736337

[2] Lemp MA Foulks GN. The definition and classification of dry eye disease: report of the Definition and Classification Subcommittee of the International Dry Eye WorkShop (2007). Ocul Surf. (2007) 5:75–92. doi: 10.1016/s1542-0124(12)70081-2 17508116

[3] Miljanović B Dana R Sullivan DA Schaumberg DA. Impact of dry eye syndrome on vision-related quality of life. Am J Ophthalmol. (2007) 143:409–15. doi: 10.1016/j.ajo.2006.11.060, PMID: 17317388 PMC1847608

[4] Talens-Estarelles C García-Marqués JV Cerviño A García-Lázaro S. Dry eye-related risk factors for digital eye strain. Eye Contact Lens. (2022) 48:410–5. doi: 10.1097/ICL.0000000000000923, PMID: 36155946

[5] Qian L Wei W. Identified risk factors for dry eye syndrome: a systematic review and meta-analysis. PLoS One. (2022) 17:e0271267. doi: 10.1371/journal.pone.0271267, PMID: 35984830 PMC9390932

[6] Australian Institute of Health and Welfare. Vision problems in older Australians. Canberra: AIHW (2005).

[7] Mohamed HB Abd El-Hamid BN Fathalla D Fouad EA. Current trends in pharmaceutical treatment of dry eye disease: a review. Eur J Pharm Sci. (2022) 175:106206. doi: 10.1016/j.ejps.2022.106206, PMID: 35568107

[8] Noble BA Loh RS MacLennan S Pesudovs K Reynolds A Bridges LR. Comparison of autologous serum eye drops with conventional therapy in a randomised controlled crossover trial for ocular surface disease. Br J Ophthalmol. (2004) 88:647–52. doi: 10.1136/bjo.2003.026211, PMID: 15090417 PMC1772131

[9] Lomas RJ Chandrasekar A Macdonald-Wallis C Kaye S Rauz S Figueiredo FC. Patient-reported outcome measures for a large cohort of serum eye drops recipients in the UK. Eye. (2021) 35:3425–32. doi: 10.1038/s41433-021-01560-8, PMID: 34531551 PMC8602237

[10] Cui D Li G Akpek EK. Autologous serum eye drops for ocular surface disorders. Curr Opin Allergy Clin Immunol. (2021) 21:493–9. doi: 10.1097/ACI.0000000000000770, PMID: 34261888

[11] Yu AT Lee GA Vincent S Shah P. Patient perceptions of autologous serum eye drops for severe dry eye disease. Clin Exp Ophthalmol. (2020) 48:1109–11. doi: 10.1111/ceo.13832, PMID: 32710460

[12] Pan Q Angelina A Marrone M Stark WJ Akpek E. Autologous serum eye drops for dry eye. Cochrane Database Syst Rev. (2017) 2:CD009327. doi: 10.1002/14651858.CD009327.pub3, PMID: 28245347 PMC5510593

[13] Franchini M Cruciani M Mengoli C Marano G Capuzzo E Pati I. Serum eye drops for the treatment of ocular surface diseases: a systematic review and meta-analysis. Blood Transfus. (2019) 17:200–9. doi: 10.2450/2019.0080-19, PMID: 31246562 PMC6596382

[14] Mondy P Brama T Fisher J Gemelli CN Chee K Keegan A. Sustained benefits of autologous serum eye drops on self-reported ocular symptoms and vision-related quality of life in Australian patients with dry eye and corneal epithelial defects. Transfus Apher Sci. (2015) 53:404–11. doi: 10.1016/j.transci.2015.11.011, PMID: 26626963

[15] Fox R Chan R Michelson J Belmont J Michelson P. Beneficial effect of artificial tears made with autologous serum in patients with keratoconjuncitivitis sicca. Arthritis Rheum. (1984) 27:459–61. doi: 10.1002/art.1780270415, PMID: 6712760

[16] Hadassah J Bhuvaneshwari N Singh D Sehgal PK. Preparation and clinical evaluation of succinylated collagen punctal plugs in dry eye syndrome: a pilot study. Ophthalmic Res. (2010) 43:185–92. doi: 10.1159/000272022, PMID: 20090392

[17] Marks DC Fisher J Mondy P Segatchian J Dennington PM. Serum eye drop preparation in Australia: current manufacturing practice. Transfus Apher Sci. (2015) 53:92–4. doi: 10.1016/j.transci.2015.05.015, PMID: 26123029

[18] van der Meer PF Verbakel SK Honohan Á Lorinser J Thurlings RM Jacobs JFM. Allogeneic and autologous serum eye drops: a pilot double-blind randomized crossover trial. Acta Ophthalmol. (2021) 99:837–42. doi: 10.1111/aos.14788, PMID: 33590715 PMC9544559

[19] Tan JCG Webb RG Marks DC. Serum growth factor stability in different eye drop packaging systems during storage. Transfus Apher Sci. (2020) 59:102608. doi: 10.1016/j.transci.2019.06.032, PMID: 31320279

[20] Hogg P Vere R Elen D Lomas R Chandrasekar A Rooney P. Validation of a closed system for dispensing serum eye drops. BMJ Open Ophthalmol. (2022) 7:A12. doi: 10.1136/bmjophth-2022-EEBA.28, PMID: 37282689 PMC11366794

[21] Harris PA Taylor R Thielke R Payne J Gonzalez N Conde JG. Research electronic data capture (REDCap)—a metadata-driven methodology and workflow process for providing translational research informatics support. J Biomed Inform. (2009) 42:377–81. doi: 10.1016/j.jbi.2008.08.010, PMID: 18929686 PMC2700030

[22] Chalmers RL Begley CG Caffery B. Validation of the 5-item dry eye questionnaire (DEQ-5): discrimination across self-assessed severity and aqueous tear deficient dry eye diagnoses. Cont Lens Anterior Eye. (2010) 33:55–60. doi: 10.1016/j.clae.2009.12.010, PMID: 20093066

[23] Keller SD Ware JE Jr Bentler PM Aaronson NK Alonso J Apolone G. Use of structural equation modeling to test the construct validity of the SF-36 health survey in ten countries: results from the IQOLA Project. International Quality of Life Assessment. J Clin Epidemiol. (1998) 51:1179–88. doi: 10.1016/S0895-4356(98)00110-3, PMID: 9817136

[24] Ware J Kosinski M Dewey J Gandek B Kisinski M Ware J. How to score and interpret single-item health status measures: a manual for users of the SF-8^™^ health survey. Lincoln, RI: QualityMetric Incorporated. (2001). 15:5.

[25] Goldstein JE Bradley C Gross AL Jackson M Bressler N Massof RW. The NEI VFQ-25C: calibrating items in the National Eye Institute visual function questionnaire-25 to enable comparison of outcome measures. Transl Vis Sci Technol. (2022) 11:10. doi: 10.1167/tvst.11.5.10, PMID: 35543680 PMC9100478

[26] Peipert JD Beaumont JL Bode R Cella D Garcia SF Hahn EA. Development and validation of the functional assessment of chronic illness therapy treatment satisfaction (FACIT TS) measures. Qual Life Res. (2014) 23:815–24. doi: 10.1007/s11136-013-0520-8, PMID: 24062239

[27] Badami KG McKellar M. Allogeneic serum eye drops: time these became the norm? Br J Ophthalmol. (2012) 96:1151–2. doi: 10.1136/bjophthalmol-2012-301668, PMID: 22539749

[28] Campos E Versura P Buzzi M Fontana L Giannaccare G Pellegrini M. Blood derived treatment from two allogeneic sources for severe dry eye associated to keratopathy: a multicentre randomised cross over clinical trial. Br J Ophthalmol. (2020) 104:1142–7. doi: 10.1136/bjophthalmol-2019-314859, PMID: 31744796

