# Supplementary material for: Patient-reported outcomes of serum eye drops manufactured from Australian blood donations and packaged using Meise vials
Source: Front Med (Lausanne). 2023 Sep 5;10:1252688. doi: 10.3389/fmed.2023.1252688 (PMC10507724; doi:10.3389/fmed.2023.1252688)
Supplement: Supplementary file 1 [file Data_Sheet_1.doc]

**Supplementary figure 1: Detailed survey questions sent to consented participants. Existing patients only completed the survey at one timepoint and new patients received the survey at baseline then 3- and 6-months post-treatment.**

**Existing Serum Eye Drop Patients Survey**

**Part 1: Demographic details and Medical History**

1. What is your date of birth? dd/mm/yyyy
2. Overall, how would you rate your health during the past 4 weeks?

Excellent Very Good Good Fair Poor Very poor

1. During the past 4 weeks, how much did physical health problems limit your usual physical activities (such as walking or climbing stairs)?

Not at all Very little Somewhat Quite a lot Could not do physical activities

1. During the past 4 weeks, how much difficulty did you have doing your daily work, both at home and away from home, because of your physical health?

None at all A little bit Some Quite a lot Could not do daily work

1. How much bodily pain have you had during the past 4 weeks?

None Very mild Mild Moderate Severe Very Severe

1. During the past 4 weeks, how much energy did you have?

Very much Quite a lot Some A little None

1. During the past 4 weeks, how much did your physical health or emotional problems limit your usual social activities with family or friends?

Not at all Very little Somewhat Quite a lot Could not do social activities

1. During the past 4 weeks, how much have you been bothered by emotional problems (such as feeling anxious, depressed, or irritable)?

Not at all Slightly Moderately Quite a lot Extremely

1. During the past 4 weeks, how much did personal or emotional problems keep you from doing your usual work, school or other daily activities?

Not at all Very little Somewhat Quite a lot Could not do daily activities

**Part 2: Symptoms and Visual Functioning**

The next set of questions will ask you about your eye symptoms. Please answer the questions honestly and to the best of your ability.

1. How long have you had your eye symptoms?

weeks months years

1. Have you had or are you currently using any other treatment (in addition to serum eye drops) for your eye condition? (please circle)

Yes No [Skip to 13]

1. If Yes, please indicate which treatment and their effectiveness of the treatment

| Treatment | Effectiveness |  |  |  |  |
| --- | --- | --- | --- | --- | --- |
|  | No help at all | Not much help | Some help | Significant help | Complete Cure |
| Artificial tears | 1 | 2 | 3 | 4 | 5 |
| Topical anti-inflammatory drops (eg cyclosporine, steroids) | 1 | 2 | 3 | 4 | 5 |
| Tear retention therapies (eg punctal plugs) | 1 | 2 | 3 | 4 | 5 |
| Other – specify: | 1 | 2 | 3 | 4 | 5 |

*Part 2.1: DEQ5*

Questions about eye discomfort:

1. During a typical day in the past month, **how often** did your eyes feel discomfort?

| Never | Rarely | Sometimes | Frequently | Constantly |
| --- | --- | --- | --- | --- |
| 0 | 1 | 2 | 3 | 4 |

1. When your eyes felt discomfort, **how intense was this feeling of discomfort** at the end of the day, within two hours of going to bed?

| Never have it | Not at all intense |  |  | Very Intense |
| --- | --- | --- | --- | --- |
| 0 | 1 | 2 | 3 | 4 |

Questions about eye dryness:

1. During a typical day in the past month, **how often** did your eyes feel dry?

| Never | Rarely | Sometimes | Frequently | Constantly |
| --- | --- | --- | --- | --- |
| 0 | 1 | 2 | 3 | 4 |

1. When your eyes felt dry, **how intense was this feeling of dryness** at the end of the day, within two hours of going to bed?

| Never have it | Not at all intense |  |  | Very Intense |
| --- | --- | --- | --- | --- |
| 0 | 1 | 2 | 3 | 4 |

Questions about watery eyes:

1. During a typical day in the past month, **how often** did your eyes look or feel excessively watery?

| Never | Rarely | Sometimes | Frequently | Constantly |
| --- | --- | --- | --- | --- |
| 0 | 1 | 2 | 3 | 4 |

Part 2.2 Wellbeing/distress

1. The next questions are about how you deal with your vision. For each of statement, please indicate how true or false each statement is for you

|  | Definitely True | Mostly True | Not Sure | Mostly False | Definitely False |
| --- | --- | --- | --- | --- | --- |
| I am often irritable because of my eyesight | 1 | 2 | 3 | 4 | 5 |
| I don’t go out of my home alone, because of my eyesight | 1 | 2 | 3 | 4 | 5 |

**Part 3: Treatment related questions (FACIT)**

These questions will ask you about how you are finding the use of serum eyedrops as a treatment. Please answer the questions honestly and to the best of your ability

| 19. Compared to what you expected, how do you rate the effectiveness of the treatment so far? | | **A lot worse**  0 | | **A little worse**  1 | | **About the same**  2 | | | **A little better**  3 | | | **A lot better**  4 | |  |
| --- | --- | --- | --- | --- | --- | --- | --- | --- | --- | --- | --- | --- | --- | --- |
| 20. Compared to what you expected, how do you rate the side effects of treatment so far? | | 0 | | 1 | | 2 | | | 3 | | | 4 | |  |
|  | **No, not at all** | | | | **Yes, to some extent** | | | **Yes, for the most part** | | | **Yes, completely** | | | |
| 21. Did your doctor(s) help you evaluate the effects of your treatment so far? | 0 | | | | 1 | | | 2 | | | 3 | | | |
| 22. Do you feel you received the treatment that was right for you? | 0 | | | | 1 | | | 2 | | | 3 | | | |
| 23. Are you satisfied with the effects of this treatment so far? | 0 | | | | 1 | | | 2 | | | 3 | | | |
| 24. Would you recommend this treatment to others with your illness? | | | **No**  0 | | | | **Maybe**  1 | | | **Yes**  2 | | |  | |
| 25. Would you choose this treatment again? | | | 0 | | | | 1 | | | 2 | | |  | |

26. How do you rate this treatment overall? **Poor Fair Good Very good Excellent**

0 1 2 3 4

**Part 4: Health service utilisation**

1. In the last 3 months after starting your eye drops, how many times have you seen the following practitioners in regards to your eye condition? Please provide your best guess

| Primary Care Physician/ GP | | | | |
| --- | --- |
| Doctor in private specialist clinic for any reason | | | | |
| Doctor in public outpatient clinic for any reason | | | | |
| Other | | | | |

**Part 5: Serum eye drop brochure information**

These questions relate to the serum eye drop brochure provided with your eyedrops

1. Did you receive the serum eyedrops brochure? [Insert Picture if applicable]

Yes No [Skip to 38]

1. **Autologous donors only**: How easy was it to understand the information in the brochure about how to prepare for your blood donation?

| Very Difficult | Difficult | Neither difficult or easy | Easy | Very Easy |
| --- | --- | --- | --- | --- |
| 1 | 2 | 3 | 4 | 5 |

1. **Autologous donors only**: How easy was it to understand the information in the brochure about what to expect when you donate?

| Very Difficult | Difficult | Neither difficult or easy | Easy | Very Easy |
| --- | --- | --- | --- | --- |
| 1 | 2 | 3 | 4 | 5 |

1. **All participants**: How easy was it to understand the information about how serum eyedrops are made?

| Very Difficult | Difficult | Neither difficult or easy | Easy | Very Easy |
| --- | --- | --- | --- | --- |
| 1 | 2 | 3 | 4 | 5 |

1. How easy was it to understand the information in the brochure about how to collect and store your serum eyedrops?

| Very Difficult | Difficult | Neither difficult or easy | Easy | Very Easy |
| --- | --- | --- | --- | --- |
| 1 | 2 | 3 | 4 | 5 |

1. How easy was it to understand the information about how to dispose of the eyedrops and the risks of the treatment?

| Very Difficult | Difficult | Neither difficult or easy | Easy | Very Easy |
| --- | --- | --- | --- | --- |
| 1 | 2 | 3 | 4 | 5 |

1. Did you access the animated video of the serum eyedrop journey either via the QR code or the provided link?

Yes No [Skip to 28]

1. How easy was it to understand the animated version of the serum eye drop journey?

| Very Difficult | Difficult | Neither difficult or easy | Easy | Very Easy |
| --- | --- | --- | --- | --- |
| 1 | 2 | 3 | 4 | 5 |

1. Is there anything that could be improved in the brochure?
   - No
   - Yes, please explain
2. Did you find the instructions on the back of the carton helpful?

Yes No

**Part 6: Serum eye drop use**

1. Are you happy with the current packaging of the eye drops?
   - Yes [Skip to 40]
   - No
2. How could we improve the current packaging?
3. How often do you use the eye drops per day?
4. How many drops do you administer each time?
5. Did you know 1 box had enough for eye drops for one month?
   - No
   - Yes
6. How many vials do you use per day?
7. Did you find it easy to open and close the vials?
   - No
   - Yes
8. Have you had to dispose of any full vials due to them being damaged?
   - No
   - Yes, if so, how many
9. Have you had to dispose of any full vials due to them expiring?
   - No
   - Yes, if so, how many
10. If you were able to use serum eye drops produced from donations given by volunteer blood donors, would you do so?

- Yes
- No, why?

**Part 7: future contact**

We may like to conduct a phone interview about your experience using the eyedrops. Would you be interested in being contacted to discuss your experience?

- - No
  - Yes, please provide best contact details

Phone Number: Email:

Thank you for participating in our survey. If you have any questions you can contact the research team on [donorresearch@redcrossblood.org.au](mailto:donorresearch@redcrossblood.org.au)

**New Serum Eye Drop Patients Survey: Baseline**

**Part 1: Demographics and medical history**

1. What is your date of birth? dd/mm/yyyy
2. Overall, how would you rate your health during the past 4 weeks?

Excellent Very Good Good Fair Poor Very poor

1. During the past 4 weeks, how much did physical health problems limit your usual physical activities (such as walking or climbing stairs)?

Not at all Very little Somewhat Quite a lot Could not do physical activities

1. During the past 4 weeks, how much difficulty did you have doing your daily work, both at home and away from home, because of your physical health?

None at all A little bit Some Quite a lot Could not do daily work

1. How much bodily pain have you had during the past 4 weeks?

None Very mild Mild Moderate Severe Very Severe

1. During the past 4 weeks, how much energy did you have?

Very much Quite a lot Some A little None

1. During the past 4 weeks, how much did your physical health or emotional problems limit your usual social activities with family or friends?

Not at all Very little Somewhat Quite a lot Could not do social activities

1. During the past 4 weeks, how much have you been bothered by emotional problems (such as feeling anxious, depressed, or irritable)?

Not at all Slightly Moderately Quite a lot Extremely

1. During the past 4 weeks, how much did personal or emotional problems keep you from doing your usual work, school or other daily activities?

Not at all Very little Somewhat Quite a lot Could not do daily activities

**Part 2: Symptoms and visual functioning**

The next set of questions will ask you about your eye symptoms. Please answer the questions honestly and to the best of your ability.

1. How long have you had your eye symptoms?

weeks months years

1. Have you had or are you currently using any other treatment (in addition to serum eye drops) for your eye condition? (please circle)

Yes No [Skip to 13]

1. If Yes, please indicate which treatment and their effectiveness of the treatment

| Treatment | Effectiveness |  |  |  |  |
| --- | --- | --- | --- | --- | --- |
|  | No help at all | Not much help | Some help | Significant help | Complete Cure |
| Artificial tears | 1 | 2 | 3 | 4 | 5 |
| Topical anti-inflammatory drops (eg cyclosporine, steroids) | 1 | 2 | 3 | 4 | 5 |
| Tear retention therapies (eg punctal plugs) | 1 | 2 | 3 | 4 | 5 |
| Other – specify: | 1 | 2 | 3 | 4 | 5 |

*Part 2.1: DEQ5*

Questions about eye discomfort:

1. During a typical day in the past month, **how often** did your eyes feel discomfort?

| Never | Rarely | Sometimes | Frequently | Constantly |
| --- | --- | --- | --- | --- |
| 0 | 1 | 2 | 3 | 4 |

1. When your eyes felt discomfort, **how intense was this feeling of discomfort** at the end of the day, within two hours of going to bed?

| Never have it | Not at all intense |  |  | Very Intense |
| --- | --- | --- | --- | --- |
| 0 | 1 | 2 | 3 | 4 |

Questions about eye dryness:

1. During a typical day in the past month, **how often** did your eyes feel dry?

| Never | Rarely | Sometimes | Frequently | Constantly |
| --- | --- | --- | --- | --- |
| 0 | 1 | 2 | 3 | 4 |

1. When your eyes felt dry, **how intense was this feeling of dryness** at the end of the day, within two hours of going to bed?

| Never have it | Not at all intense |  |  | Very Intense |
| --- | --- | --- | --- | --- |
| 0 | 1 | 2 | 3 | 4 |

Questions about watery eyes:

1. During a typical day in the past month, **how often** did your eyes look or feel excessively watery?

| Never | Rarely | Sometimes | Frequently | Constantly |
| --- | --- | --- | --- | --- |
| 0 | 1 | 2 | 3 | 4 |

*Part 2.2 Wellbeing/distress*

1. The next questions are about how you deal with your vision. For each of statement, please indicate how true or false each statement is for you

|  | Definitely True | Mostly True | Not Sure | Mostly False | Definitely False |
| --- | --- | --- | --- | --- | --- |
| I am often irritable because of my eyesight | 1 | 2 | 3 | 4 | 5 |
| I don’t go out of my home alone, because of my eyesight | 1 | 2 | 3 | 4 | 5 |

**Part 3: Health service utilisation**

1. In the last 3 months prior to starting your eye drops, how many times have you seen the following practitioners in regards to your eye condition? Please provide your best guess

| Primary Care Physician/ GP | | | | |
| --- | --- |
| Doctor in private specialist clinic for any reason | | | | |
| Doctor in public outpatient clinic for any reason | | | | |
| Other, specify | | | | |

Thank you for participating in our survey. **You will receive the next survey from us approximately 3 months after you have started your drops.** If you have any questions you can contact the research team on [donorresearch@redcrossblood.org.au](mailto:donorresearch@redcrossblood.org.au)

**Serum Eye Drop Patients Survey: 3 month**

**Part 1: Demographics and medical history**

1. What is your date of birth? dd/mm/yyyy
2. Overall, how would you rate your health during the past 4 weeks?

Excellent Very Good Good Fair Poor Very poor

1. During the past 4 weeks, how much did physical health problems limit your usual physical activities (such as walking or climbing stairs)?

Not at all Very little Somewhat Quite a lot Could not do physical activities

1. During the past 4 weeks, how much difficulty did you have doing your daily work, both at home and away from home, because of your physical health?

None at all A little bit Some Quite a lot Could not do daily work

1. How much bodily pain have you had during the past 4 weeks?

None Very mild Mild Moderate Severe Very Severe

1. During the past 4 weeks, how much energy did you have?

Very much Quite a lot Some A little None

1. During the past 4 weeks, how much did your physical health or emotional problems limit your usual social activities with family or friends?

Not at all Very little Somewhat Quite a lot Could not do social activities

1. During the past 4 weeks, how much have you been bothered by emotional problems (such as feeling anxious, depressed, or irritable)?

Not at all Slightly Moderately Quite a lot Extremely

1. During the past 4 weeks, how much did personal or emotional problems keep you from doing your usual work, school or other daily activities?

Not at all Very little Somewhat Quite a lot Could not do daily activities

**Part 2: Symptoms and visual functioning**

The next set of questions will ask you about your eye symptoms. Please answer the questions honestly and to the best of your ability.

*Part 2.1: DEQ5*

Questions about eye discomfort:

1. During a typical day in the past month, **how often** did your eyes feel discomfort?

| Never | Rarely | Sometimes | Frequently | Constantly |
| --- | --- | --- | --- | --- |
| 0 | 1 | 2 | 3 | 4 |

1. When your eyes felt discomfort, **how intense was this feeling of discomfort** at the end of the day, within two hours of going to bed?

| Never have it | Not at all intense |  |  | Very Intense |
| --- | --- | --- | --- | --- |
| 0 | 1 | 2 | 3 | 4 |

Questions about eye dryness:

1. During a typical day in the past month, **how often** did your eyes feel dry?

| Never | Rarely | Sometimes | Frequently | Constantly |
| --- | --- | --- | --- | --- |
| 0 | 1 | 2 | 3 | 4 |

1. When your eyes felt dry, **how intense was this feeling of dryness** at the end of the day, within two hours of going to bed?

| Never have it | Not at all intense |  |  | Very Intense |
| --- | --- | --- | --- | --- |
| 0 | 1 | 2 | 3 | 4 |

Questions about watery eyes:

1. During a typical day in the past month, **how often** did your eyes look or feel excessively watery?

| Never | Rarely | Sometimes | Frequently | Constantly |
| --- | --- | --- | --- | --- |
| 0 | 1 | 2 | 3 | 4 |

*Part 2.2 Wellbeing/distress*

1. The next questions are about how you deal with your vision. For each of statement, please indicate how true or false each statement is for you based on the last 3 months

|  | Definitely True | Mostly True | Not Sure | Mostly False | Definitely False |
| --- | --- | --- | --- | --- | --- |
| I am often irritable because of my eyesight | 1 | 2 | 3 | 4 | 5 |
| I don’t go out of my home alone, because of my eyesight | 1 | 2 | 3 | 4 | 5 |

**Part 3: Treatment related questions (FACIT)**

These questions will ask you about how you are finding the use of serum eyedrops as a treatment. Please answer the questions honestly and to the best of your ability

| 16. Compared to what you expected, how do you rate the effectiveness of the treatment so far? | **A lot worse**  0 | | | **A little worse**  1 | | **About the same**  2 | | | **A little better**  3 | | | **A lot better**  4 | |  |
| --- | --- | --- | --- | --- | --- | --- | --- | --- | --- | --- | --- | --- | --- | --- |
| 17. Compared to what you expected, how do you rate the side effects of treatment so far? | 0 | | | 1 | | 2 | | | 3 | | | 4 | |  |
|  | | **No, not at all** | | | **Yes, to some extent** | | | **Yes, for the most part** | | | **Yes, completely** | | | |
| 18. Did your doctor(s) help you evaluate the effects of your treatment so far? | | 0 | | | 1 | | | 2 | | | 3 | | | |
| 19. Do you feel you received the treatment that was right for you? | | 0 | | | 1 | | | 2 | | | 3 | | | |
| 20. Are you satisfied with the effects of this treatment so far? | | 0 | | | 1 | | | 2 | | | 3 | | | |
| 21. Would you recommend this treatment to others with your illness? | | | **No**  0 | | | | **Maybe**  1 | | | **Yes**  2 | | |  | |
| 22. Would you choose this treatment again? | | | 0 | | | | 1 | | | 2 | | |  | |

**Poor Fair Good Very Good Excellent**

23. How do you rate this treatment overall? 0 1 2 3 4

**Part 4: Health service utilisation**

1. In the last 3 months after starting your eye drops, how many times have you seen the following practitioners in regards to your eye condition? Please provide your best guess

| Primary Care Physician/ GP | | | | |
| --- | --- |
| Doctor in private specialist clinic for any reason | | | | |
| Doctor in public outpatient clinic for any reason | | | | |
| Other | | | | |

**Part 5: Serum eye drop brochure information**

These questions relate to the serum eye drop brochure provided with your eyedrops

1. Did you receive the serum eyedrops brochure? [Insert Picture if applicable]

Yes No [Skip to 34]

1. **Autologous donors only**: How easy was it to understand the information in the brochure about how to prepare for your blood donation?

| Very Difficult | Difficult | Neither difficult or easy | Easy | Very Easy |
| --- | --- | --- | --- | --- |
| 1 | 2 | 3 | 4 | 5 |

1. **Autologous donors only**: How easy was it to understand the information in the brochure about what to expect when you donate?

| Very Difficult | Difficult | Neither difficult or easy | Easy | Very Easy |
| --- | --- | --- | --- | --- |
| 1 | 2 | 3 | 4 | 5 |

1. **All participants**: How easy was it to understand the information about how serum eyedrops are made?

| Very Difficult | Difficult | Neither difficult or easy | Easy | Very Easy |
| --- | --- | --- | --- | --- |
| 1 | 2 | 3 | 4 | 5 |

1. How easy was it to understand the information in the brochure about how to collect and store your serum eyedrops?

| Very Difficult | Difficult | Neither difficult or easy | Easy | Very Easy |
| --- | --- | --- | --- | --- |
| 1 | 2 | 3 | 4 | 5 |

1. How easy was it to understand the information about how to dispose of the eyedrops and the risks of the treatment?

| Very Difficult | Difficult | Neither difficult or easy | Easy | Very Easy |
| --- | --- | --- | --- | --- |
| 1 | 2 | 3 | 4 | 5 |

1. Did you access the animated video of the serum eyedrop journey either via the QR code or the provided link?

Yes No [Skip to 28]

1. How easy was it to understand the animated version of the serum eye drop journey?

| Very Difficult | Difficult | Neither difficult or easy | Easy | Very Easy |
| --- | --- | --- | --- | --- |
| 1 | 2 | 3 | 4 | 5 |

1. Is there anything that could be improved in the brochure?
   - No
   - Yes, please explain
2. Did you find the instructions on the back of the eye drop box helpful?

- Yes
- No

**Part 6: Serum eye drop use**

1. Are you happy with the current packaging of the eye drops?
   - Yes
   - No
2. How could we improve the current packaging?
3. How many times a day do you use the eye drops?
4. How many drops do you use administer each time?
5. Did you know 1 box had enough for eye drops for one month?

- No
- Yes

1. How many vials do you use per day?
2. Did you find it easy to open and close the vials?

- No
- Yes

1. Have you had to dispose of any full vials due to them being damaged?

- No
- Yes, if so, how many

1. Have you had to dispose of any full vials due to them expiring?

- No
- Yes, if so, how many

1. If you were able to use serum eye drops produced from donations given by volunteer blood donors, would you do so?[ASED patients only]

- Yes
- No, why?

**Part 7: future contact**

We may like to conduct a phone interview about your experience using the eyedrops. Would you be interested in being contacted to discuss your experience?

- - No
  - Yes, please provide best contact details

Phone Number: Email:

Thank you for participating in our survey. **You will receive the last survey from us in approximately 3 months’ time.** If you have any questions you can contact the research team on [donorresearch@redcrossblood.org.au](mailto:donorresearch@redcrossblood.org.au)

**Serum Eye Drop Patients Survey: 6 month**

**Part 1: Demographics and medical history**

1. What is your date of birth? dd/mm/yyyy
2. Overall, how would you rate your health during the past 4 weeks?

Excellent Very Good Good Fair Poor Very poor

1. During the past 4 weeks, how much did physical health problems limit your usual physical activities (such as walking or climbing stairs)?

Not at all Very little Somewhat Quite a lot Could not do physical activities

1. During the past 4 weeks, how much difficulty did you have doing your daily work, both at home and away from home, because of your physical health?

None at all A little bit Some Quite a lot Could not do daily work

1. How much bodily pain have you had during the past 4 weeks?

None Very mild Mild Moderate Severe Very Severe

1. During the past 4 weeks, how much energy did you have?

Very much Quite a lot Some A little None

1. During the past 4 weeks, how much did your physical health or emotional problems limit your usual social activities with family or friends?

Not at all Very little Somewhat Quite a lot Could not do social activities

1. During the past 4 weeks, how much have you been bothered by emotional problems (such as feeling anxious, depressed, or irritable)?

Not at all Slightly Moderately Quite a lot Extremely

1. During the past 4 weeks, how much did personal or emotional problems keep you from doing your usual work, school or other daily activities?

Not at all Very little Somewhat Quite a lot Could not do daily activities

**Part 2: Symptoms and visual functioning**

The next set of questions will ask you about your eye symptoms. Please answer the questions honestly and to the best of your ability.

*Part 2.1: DEQ5*

Questions about eye discomfort:

1. During a typical day in the past month, **how often** did your eyes feel discomfort?

| Never | Rarely | Sometimes | Frequently | Constantly |
| --- | --- | --- | --- | --- |
| 0 | 1 | 2 | 3 | 4 |

1. When your eyes felt discomfort, **how intense was this feeling of discomfort** at the end of the day, within two hours of going to bed?

| Never have it | Not at all intense |  |  | Very Intense |
| --- | --- | --- | --- | --- |
| 0 | 1 | 2 | 3 | 4 |

Questions about eye dryness:

1. During a typical day in the past month, **how often** did your eyes feel dry?

| Never | Rarely | Sometimes | Frequently | Constantly |
| --- | --- | --- | --- | --- |
| 0 | 1 | 2 | 3 | 4 |

1. When your eyes felt dry, **how intense was this feeling of dryness** at the end of the day, within two hours of going to bed?

| Never have it | Not at all intense |  |  | Very Intense |
| --- | --- | --- | --- | --- |
| 0 | 1 | 2 | 3 | 4 |

Questions about watery eyes:

1. During a typical day in the past month, **how often** did your eyes look or feel excessively watery?

| Never | Rarely | Sometimes | Frequently | Constantly |
| --- | --- | --- | --- | --- |
| 0 | 1 | 2 | 3 | 4 |

*Part 2.2 Wellbeing/distress*

1. The next questions are about how you deal with your vision. For each of statement, please indicate how true or false each statement is for you

|  | Definitely True | Mostly True | Not Sure | Mostly False | Definitely False |
| --- | --- | --- | --- | --- | --- |
| I am often irritable because of my eyesight | 1 | 2 | 3 | 4 | 5 |
| I don’t go out of my home alone, because of my eyesight | 1 | 2 | 3 | 4 | 5 |

**Part 3: Treatment related questions (FACIT)**

These questions will ask you about how you are finding the use of serum eyedrops as a treatment. Please answer the questions honestly and to the best of your ability

| 16. Compared to what you expected, how do you rate the effectiveness of the treatment so far? | **A lot worse**  0 | **A little worse**  1 | **About the same**  2 | **A little better**  3 | **A lot better**  4 |
| --- | --- | --- | --- | --- | --- |
| 17. Compared to what you expected, how do you rate the side effects of treatment so far? | 0 | 1 | 2 | 3 | 4 |

**No, not at all**

**Yes, to some extent**

**Yes, for the most part**

**Yes, completely**

| 18. Did your doctor(s) help you evaluate the effects of your treatment so far? | 0 | 1 | 2 | 3 |
| --- | --- | --- | --- | --- |
| 19. Do you feel you received the treatment that was right for you? | 0 | 1 | 2 | 3 |
| 20. Are you satisfied with the effects of this treatment so far? | 0 | 1 | 2 | 3 |

| 21. Would you recommend this treatment to others with your illness? | **No**  0 | **Maybe**  1 | **Yes**  2 |
| --- | --- | --- | --- |
| 22. Would you choose this treatment again? | 0 | 1 | 2 |

23. How do you rate this treatment overall? **Poor Fair Good Very good Excellent**

0 1 2 3 4

**Part 4: Health service utilisation**

24. In the last 3 months, how many times have you seen the following practitioners in regards to your eye condition? Please provide your best guess

| Primary Care Physician/ GP | | | | |
| --- | --- |
| Doctor in private specialist clinic for any reason | | | | |
| Doctor in public outpatient clinic for any reason | | | | |
| Other | | | | |

**Part 5: Serum eye drop use**

Thinking about the last 3 months,

1. How many times a day do you use the eye drops?
2. How many drops do you use administer each time?
3. How many vials do you use per day?
4. Have you had to dispose of any full vials due to them being damaged?
   - No
   - Yes, if so, how many

29. Have you had to dispose of any full vials due to them expiring?

- - No
  - Yes, if so, how many

Thank you for participating in our survey. If you have any questions you can contact the research team on [donorresearch@redcrossblood.org.au](mailto:donorresearch@redcrossblood.org.au)

**Supplementary figure 2. Structure telephone interview**

[Existing Auto Patients]: Do you have any comments on the new packaging compared to the old packaging for your eyedrops?

[New/PT Patients]: Do you have any comments on the packaging used for the eyedrops?

[Everyone]: How did you find getting the drops out of the dropper? If difficult, did you come up with any solutions to make it easier for you?

*Prompt: Issues with getting drops out.*

[Everyone]: How did you store the vial once you opened it?

[Everyone]: How long did you use your open vial?

[Everyone]: Did you have to discard eyedrop vials, if so why?

*Prompt: Expiry/not stored correctly*

[Everyone]: How did you find the instructions in the brochure and on the vials? Was it easy for you to read these? Is there anything that could be improved?

**Allogeneic eye drops**

[Auto patients]: Were you aware eye serum drops can be produced from donations given by volunteer blood donors?

What do you know about eye drops that come from other donors?

[Auto patients]: Some patients are already using eye drops that come from other donors. Is there any information you would you like to know before changing over to donor eyedrops?

[Auto patients]: Would you have any concerns if you switched from donating your own eye drops to receiving donor eye drops? If yes, can you tell me a bit about your concerns

[PT Existing patients (prior auto donors)]: Did you have any concerns when you switched from donating your own eye drops (autologous) to receiving donor eye drops (allogenic)?

*Prompt: Any differences between the drops*

[PT Existing patients (prior auto donors)]: How do the donor eye drops compare to your own eye drops?

*Prompt: What packaging did your own eye drops come in MEISE vs tubing*

[PT patients]: What would be the impact if you were unable to access drops donated by a volunteer donor?

**Service**

[Auto patients]: How did you find the experience of going into a donor centre to donate?

*Prompt for difficulty reading forms, getting to a donor centre.*

[Auto patients]: Did you need assistance to get to the donor centre?

[Auto patients]: Did you have any concerns about donating blood for your eyedrops? If response is negative, do you think it would be easier if you could use eyedrops from other donors?

[PT patients]: How was your experience being referred to get donor eye drops?

*Prompt: Knowledge of being referred for PT from ophthalmologist*

[Everyone]: What was your experience like when you went to collect your eyedrops?

*Prompt for difficulty getting there, communications from ophthalmologist and Lifeblood*

*[Everyone]: Did you receive adequate information from your ophthalmologist about how often to use the drops?*

**Quality of life**

[Everyone]: Do you travel with the drops and how do you store them?

[Everyone]: Would you still use these eye drops if you had to cover some of the cost?

[Everyone]: Has using the eye drops had an impact on your life, such as on everyday activities? If yes, can you tell me a bit about this?

Is there anything else you would like to tell us about using eye drops?
